# Development and validation of a GT‐seq panel for genetic monitoring in a threatened species using minimally invasive sampling

**DOI:** 10.1002/ece3.11321

**Published:** 2024-05-20

**Authors:** Molly J. Garrett, Stacey A. Nerkowski, Shannon Kieran, Nathan R. Campbell, Soraia Barbosa, Courtney J. Conway, Paul A. Hohenlohe, Lisette P. Waits

**Affiliations:** ^1^ Department of Fish and Wildlife Sciences, College of Natural Resources University of Idaho Moscow Idaho USA; ^2^ GTseek LLC Twin Falls Idaho USA; ^3^ U.S. Geological Survey, Idaho Cooperative Fish & Wildlife Research Unit University of Idaho Moscow Idaho USA; ^4^ Department of Biological Sciences, College of Science University of Idaho Moscow Idaho USA

**Keywords:** conservation genomics, northern Idaho ground squirrel, single nucleotide polymorphisms, SNPs, targeted amplicon sequencing, *Urocitellus brunneus*

## Abstract

Minimally invasive samples are often the best option for collecting genetic material from species of conservation concern, but they perform poorly in many genomic sequencing methods due to their tendency to yield low DNA quality and quantity. Genotyping‐in‐thousands by sequencing (GT‐seq) is a powerful amplicon sequencing method that can genotype large numbers of variable‐quality samples at a standardized set of single nucleotide polymorphism (SNP) loci. Here, we develop, optimize, and validate a GT‐seq panel for the federally threatened northern Idaho ground squirrel (*Urocitellus brunneus*) to provide a standardized approach for future genetic monitoring and assessment of recovery goals using minimally invasive samples. The optimized panel consists of 224 neutral and 81 putatively adaptive SNPs. DNA collected from buccal swabs from 2016 to 2020 had 73% genotyping success, while samples collected from hair from 2002 to 2006 had little to no DNA remaining and did not genotype successfully. We evaluated our GT‐seq panel by measuring genotype discordance rates compared to RADseq and whole‐genome sequencing. GT‐seq and other sequencing methods had similar population diversity and *F*_ST_ estimates, but GT‐seq consistently called more heterozygotes than expected, resulting in negative *F*_IS_ values at the population level. Genetic ancestry assignment was consistent when estimated with different sequencing methods and numbers of loci. Our GT‐seq panel is an effective and efficient genotyping tool that will aid in the monitoring and recovery of this threatened species, and our results provide insights for applying GT‐seq for minimally invasive DNA sampling techniques in other rare animals.

## Graphical abstract

Here, we develop, optimize, and validate a GT‐seq panel for the federally threatened northern Idaho ground squirrel (*Urocitellus brunneus*) to provide a standardized approach for future genetic monitoring and assessment of recovery goals using minimally invasive samples. We evaluated our GT‐seq panel by measuring genotype discordance rates compared to RADseq and whole‐genome sequencing. Our GT‐seq panel is an effective and efficient genotyping tool that will aid in the monitoring and recovery of this threatened species, and our results provide insights for applying GT‐seq for minimally invasive DNA sampling techniques in other rare animals.

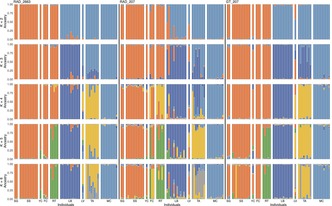

## 1 INTRODUCTION

Rapid advancements of genomic sequencing technologies have provided increased resolution and identification of neutral and adaptive genomic variants, which has improved our ability to monitor and manage wildlife populations and potentially mitigate the effects of environmental change and biodiversity loss (Hohenlohe et al., 2021; Walters & Schwartz, 2020). Genetic monitoring studies investigate both demographic and genetic parameters across time, often with noninvasive or minimally invasive samples (Schwartz et al., 2007). Noninvasive samples are left behind by an organism and are collected without having to catch, disturb, or sometimes even observe individuals, while minimally invasive samples are collected often through capture but via less invasive methods than blood or tissue extraction (Beja‐Pereira et al., 2009; Carroll et al., 2018; Taberlet et al., 1999; Waits & Paetkau, 2005). Due to their tendency to yield low DNA quality and quantity, noninvasive and minimally invasive samples are not the best options for many next‐generation sequencing approaches (Andrews et al., 2018; but see Russello et al., 2015). Restriction site‐association DNA sequencing (RADseq) is one of the most common next‐generation sequencing techniques used for single nucleotide polymorphism (SNP) discovery and genotyping of non‐model organisms. RADseq targets a subset of the genome at a greater depth of coverage per locus than whole genome sequencing techniques, which is beneficial for genotyping large numbers of samples (Andrews et al., 2016). However, RADseq requires high‐molecular‐weight genomic DNA (Graham et al., 2015), which may be limiting if samples can only be collected through non‐invasive or minimally invasive techniques. Therefore, targeted amplicon sequencing is one of several new techniques that provide powerful tools for SNP genotyping for low‐quality samples. Amplicon sequencing generates PCR products using template‐specific primers, which can have a high success rate for low‐quality samples (Andrews et al., 2018).

Genotyping‐in‐thousands by sequencing (GT‐seq; Campbell et al., 2015) is a type of amplicon sequencing that involves the development of multiplexed PCR primers. These primers target 50 to 100s of preidentified loci that are informative for a specific suite of conservation objectives. Compared to other genotyping‐by‐sequencing methods, GT‐seq is cost effective and while originally designed on tissue samples (Campbell et al., 2015), it has worked on minimally and non‐invasive samples. For example, GT‐seq panels have been created and used on polar bear (*Ursus maritimus*) fecal samples (Hayward et al., 2022), western‐rattlesnake (*Crotalus oreganus*) cloacal swabs and skin sheds (Schmidt et al., 2020), and Sitka black‐tailed deer (*Odocoileus hemionus sitkensis*) hair and fecal samples (Burgess et al., 2022).

GT‐seq panels require the previous identification of the loci of interest, generally through RAD sequencing or other approaches using high quality DNA samples. The samples used in primer and panel development should be chosen to avoid ascertain bias and to be representative of the populations in which the panel will be used, which sometimes requires that data from lower‐quality samples are also used in panel development. Additionally, an important step in panel validation is to estimate and minimize genotype discordance between GT‐seq and the genotyping performed to identify the loci for the GT‐seq panel. Studies that compared genotypes for the same individuals at the same loci generated with both RADseq and GT‐seq report discordance rates ranging from 1.77% to 3.40% (Hayward et al., 2022; Schmidt et al., 2020; Setzke et al., 2021).

Northern Idaho ground squirrels (*Urocitellus brunneus*, hereafter NIDGS) are endemic to Idaho and federally listed as a threatened species (USFWS, 2003) with an estimated population size of 2000–3000 individuals (Wagner & Evans Mack, 2020). NIDGS occupy xeric meadows and small rocky outcroppings within coniferous forests of central Idaho. They persist within only a small fraction of their former range (Helmstetter et al., 2021) and numerous causes have been proposed to explain their rarity (Sherman & Runge, 2002; Suronen & Newingham, 2013; USFWS, 2003; Yensen & Dyni, 2020). In 2003, the United States Fish and Wildlife Service (USFWS) produced a NIDGS recovery plan that delineated actions and objectives to recover and protect the species (USFWS, 2003). One objective of the recovery plan was to more accurately define NIDGS metapopulation structure and conservation units (sensu Funk et al., 2012). The plan outlines the need to understand connectivity among metapopulations (occupied sites) and the landscape factors that impede or facilitate gene flow, as well as an improved estimate of effective population size (*N*_e_).

NIDGS are semi‐colonial and occur in distinct sites interspersed within a matrix of unoccupied areas. Many of these occupied sites have been genetically sampled since 2002, but due to the threatened status of the species, managers require less stressful, minimally invasive genetic sampling techniques including plucking hair and taking buccal swabs. Hair samples collected in 2002 and 2006 were used to evaluate genetic diversity and gene flow based on mitochondrial DNA sequencing and nuclear DNA microsatellites (Garner et al., 2005; Hoisington‐Lopez et al., 2012; Zero et al., 2017). Buccal swabs collected in 2016 were sequenced with RADseq (Barbosa et al., 2021). This was the first application of genomic sequencing to NIDGS samples, which provided novel information about population structure and neutral and adaptive genetic variation across the NIDGS sites. For instance, six genetic groups were identified among 13 sampling areas using neutral loci and three distinct groups were identified using adaptive loci. Elevation appeared to be the main driver of adaptive differentiation between sites, as SNPs were associated with environmental variables such as slope, ridges, and peaks (Barbosa et al., 2021). Further, gene ontology enrichment analyses of adaptive loci found no evidence for enrichment of specific cellular components, molecular functions, or biological processes (Barbosa et al., 2021). However, this study had small sample sizes for some of the sampled sites because many of the minimally invasive samples did not produce sufficient DNA quality or quantity for RADseq. Thus, new SNP analyses that incorporate all previously sampled populations of NIDGS would greatly improve the inferences possible when assessing genetic diversity, gene flow among extant populations, and differentiation and adaptive differences among populations.

The main objective of this study was to develop a GT‐seq panel for genetic monitoring of this rare federally threatened ground squirrel using minimally invasive samples. Neutral and putatively adaptive SNPs identified through the previous RADseq study (Barbosa et al., 2021) were selected for the GT‐seq panel, which will be applied to assess objectives set by the USFWS species recovery plan for the NIDGS. The specific goals of this study were to: (1) develop and optimize a set of SNP loci for a GT‐seq panel, (2) validate the panel by comparing against RADseq and whole genome sequencing (WGS) data, and (3) evaluate the factors that produce genotype mismatches between sequencing methods.

## 2 METHODS

### 2.1 Sample collection and DNA extraction

**FIGURE 1:**
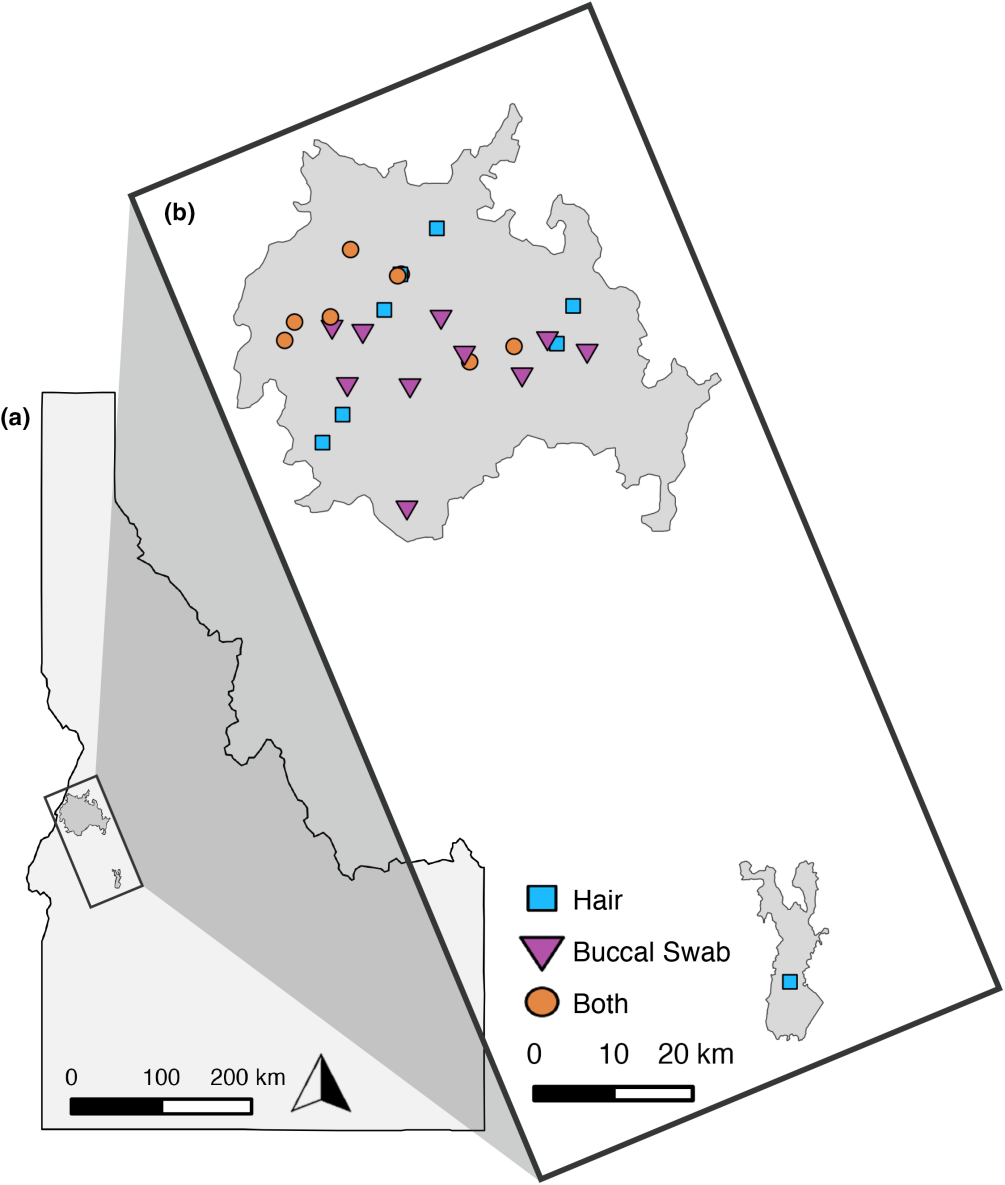
Sample distribution of hair and buccal swab samples from NIDGS in central Idaho. (a) Outline of Idaho with range of NIDGS in dark gray. (b) Detail of NIDGS range with sample types collected from each site denoted by color and shape. NIDGS range obtained from https://ecos.fws.gov/ecp/species/2982.

As part of larger, ongoing studies on habitat use and species delimitation, we extracted DNA from a total of 1086 samples, from hair, buccal swab, or tissue samples collected from NIDGS captured at occupied sites spanning two decades (Table S1). Sample collection periods were 2002–2006 (Garner et al., 2005; Hoisington‐Lopez et al., 2012), 2016 (Barbosa et al., 2021), and 2020. Some NIDGS sites were sampled during each collection period (Figure 1, Table S1), with the 2020 collection focused on obtaining samples from sites that had never been genetically sampled. Across all collection periods, Tomahawk live traps were baited and placed near burrows or logs at regularly spaced intervals within occupied sites. All NIDGS were trapped and handled following protocols and procedures approved by the University of Idaho Animal Care and Use Committee [IACUC #2006‐35 (Garner et al., 2005; Hoisington‐Lopez et al., 2012) and #2015‐53 (Barbosa et al., 2021)]. During 2002–2006, we plucked approximately 50–60 hairs from the tail of each squirrel, and we stored the hairs in an envelope with silica until DNA extraction. During 2016–2020, we swabbed the inside of each captured squirrel's cheek with a sterile cotton swab (Lakewood Biomedical) to collect epithelial cells, which was repeated five times per individual. All replicate buccal swabs per individual were preserved in the same tube in Qiagen ATL buffer until DNA extraction. Separate from the above long‐term dataset, 28 tissue samples were opportunistically collected since 2004, mostly through road‐kill collections. Twenty‐one of these tissue samples were sequenced with whole genome sequencing and the GT‐seq panel to validate the data produced from the panel but were not included in the population genetic analyses, because they were collected outside of our study's temporal range.

We used Qiagen Blood and Tissue extraction kits (Qiagen, Inc.) to extract DNA from hair, buccal swab, and tissue samples using standard protocols (Barbosa et al., 2021; Garner et al., 2005; Hoisington‐Lopez et al., 2012). The hair and buccal swab samples were extracted in the low quantity DNA facility with no PCR products or concentrated DNA. We evaluated DNA concentration of 207 hair and 454 buccal swab extracts with Qubit fluorometer quantification (Thermo Fisher Scientific). We further investigated the DNA quality and quantity of the hair extracts through gel electrophoresis (*N* = 30), genomic DNA (gDNA) fragment analysis (*N* = 9), and through the amplification of one microsatellite locus (GS17 145–167 bp, *N* = 53).

### 2.2 SNP selection and primer design

We used all 131 putatively adaptive loci that had been identified through partial redundancy analysis (pRDA) (Barbosa et al., 2021) for primer development to ensure we captured all identified adaptive loci for future analyses. Briefly, pRDA is a multivariate approach to estimate genotype–environment associations while accounting for population structure. To select neutral GT‐seq candidate SNPs, we filtered from the 2663 neutral NIDGS SNPs identified by Barbosa et al. (2021) using the following parameters: <0.4 missing data, >0.1 expected heterozygosity, and 1 SNP per scaffold (3475 total scaffolds, 3444 neutral only scaffolds). This generated 510 neutral loci for panel optimization. GTseek LLC (https://gtseek.com/) designed the GT‐seq primers for all candidate SNPs using the consensus sequence from the RADseq data from Barbosa et al. (2021) as flanking sequence around targeted SNPs. Primers were designed to be 18–24 bases in length with an annealing temperature range between 58 and 63°C before the addition of Illumina adapter sequences. Designed primer sets were then further filtered if stable hairpin structures had melting temperatures above 50°C or if primer heterodimers were predicted to form with other primers. Primers were excluded if they reported annealing temperatures to other primer sequences of 50°C or higher. As a final filtering step, primers were excluded if there were any binding sites within any of the expected amplicon sequences.

### 2.3 GT‐seq library preparation and panel development

We first produced one library following the standard GT‐seq library protocol in Campbell et al. (2015), but this library was unsuccessful. For all of the following libraries, we followed a low‐quality DNA GT‐seq protocol modified from Campbell et al. (2015) as follows: the PCR cocktail for PCR amplification of target loci included 2 μL of input gDNA for buccal swabs and 3 μL for hair samples, 3.5 μL of Qiagen Plus multiplex master mix, and 1.5 μL of pooled primer mix (final primer concentration at each locus≈160 nM). Thermal cycling was conducted in 96‐well plates, and cycles in PCR1 were increased from 10 to 16 cycles: [95°C – 15 min; 5 cycles (95°C – 30 s, 5% ramp down to 57°C – 30 s, 72°C – 2 min); 16 cycles (95°C – 30 s, 65°C – 20 s, and72°C – 30 s) 4°C hold]. The product of PCR1 was diluted 10‐fold, and 3 μL of diluted PCR1 product was transferred to a new 96‐well plate for PCR2. With a multichannel pipette, 1 μL of 10 μM well‐specific i5 tag primers were added, and 1 μL of plate‐specific tagging primers and 5 μL of Qiagen Plus multiplex master mix were added to each well. Thermal cycling conditions for PCR2 were [95°C – 15 min; 10 cycles (98°C – 10 s, 65°C – 30 s, 72°C – 30 s); 72°C – 5 min, 4°C hold]. After PCR2, only libraries with buccal swab samples were normalized using the Just‐a‐Plate™ 96 PCR Normalization and Purification kit (Charm Biotech LLC) according to the manufacturer's instructions. Next, 10 μL of each sample per 96‐well plate was pooled into a 1.5 mL Eppendorf tube. We then performed a two‐step purification with Agencourt® AMPure® XP magnetic beads to remove smaller, unwanted fragments below 150 bp and above 300 bp. After mixing and placement on a magnetic rack, the supernatant was discarded, and the immobilized beads were washed twice with 70% ethanol. The purified libraries were eluted with 30 μL of low TE.

Two rounds of optimization determined which primers would be included in the panel. The first optimization used samples that were sequenced with RADseq in Barbosa et al. (2021) while the second round included samples from the RADseq study and new samples that had not been genotyped before and had high DNA concentrations. After the first and second optimizations, primers were removed based on output plots from *GTseq_SeqTest.pl* and *GTseq_Primer‐Interaction‐Test.pl* (https://github.com/GTseq). Output plots from these scripts reported primers responsible for primer hetero‐dimer artifacts, proper amplicon targets, and primer sets that produced no sequences. Primers were omitted if they had no amplification or were otherwise negatively affecting genotyping success or target capture rates (e.g., if they were over‐abundant or had off target amplification).

A third round of optimization determined the concentration of each primer. Using the candidate primer set defined after the second optimization, two formulations were created with varying concentrations of each primer. The first was a standard primer mix with equal concentrations of each primer (following Campbell et al., 2015), with 160 nM per primer in the pooled primer mix. The second was a varied primer concentration mix, where 80 nM was used for primers that performed in the top 25% of the second optimization, 320 nM was used for primers that performed in the bottom 25% of the second optimization, and 160 nM for all other primers. Both primer mixes were applied to the same high‐quality samples.

GT‐seq libraries were sequenced using a single‐read 118 bp cycle on Illumina NovaSeq SP at the University of Oregon Genomics and Cell Characterization Core Facility (G3CF) and a paired‐end 75 bp cycle on Illumina NextSeq at Seqmatic LLC (San Francisco, CA). We sequenced 13 libraries: five of hair extracts only, six of buccal swab extracts only, one mixed of hair and buccal swab extracts, and one of tissue extracts only. The GT‐seq pipeline on GitHub (https://github.com/GTseq) was used to process raw sequence data, and only the forward sequencing reads from the paired‐end data were genotyped to match the single‐read data. Briefly, the *GTseq_Genotyper_v2.pl* perl script uses in silico probe sequences to count the occurrence of each allele within individual fastq files. Genotypes for each locus are created by the ratio of allele 1 to allele 2, where allele ratios >10 are homozygous for allele 1, ratios <0.1 are homozygous for allele 2, and ratios between 0.2 and 5.0 are heterozygous. Loci with <10 reads are not genotyped (Campbell et al., 2015). This genotyping method will be referred to as the allele ratio (AR) pipeline.

### 2.4 Tissue whole genome sequencing and genotyping

We sequenced 21 tissue samples using whole‐genome resequencing protocols. Illumina library preparation was undertaken by the University of Idaho Genomics and Bioinformatics Resources Core (GBRC; Moscow, Idaho) using the Illumina Nextera library preparation kit following manufacturer's protocols. Samples were PE150‐sequenced on a single S4 lane of a Novaseq 4000X by the University of Oregon G3CF. Samples averaged 114 M reads/sample before filtering.

For this project, samples were genotyped exclusively at the loci used for GT‐seq sequencing. First, samples were aligned to the same genome used when genotyping the RADseq dataset, downloaded from NCBI (SpeTri2.0, accession #GCF_000236235.1, accessed 7/09/2023). Sequences were aligned using *bwa mem* (Li & Durbin, 2010). Alignments were sorted and filtered to remove duplicates and exclude improperly paired reads. Genotypes were called in angsd 0.981 (Korneliussen et al., 2014) using the following settings: ‐minDepth 126 (avg 6×), ‐minInds 16 (76%), ‐minMaf 0.05, ‐postCutoff 0.95 ‐snp‐pval 1e‐6, ‐minQ 20, ‐minMapQ 10. A sites file with the positions of the GT‐seq loci was passed to angsd along with the list of input bamfiles.

### 2.5 Genotyping discordance

To validate the GT‐seq panel, we compared two sets of samples: buccal swabs that were genotyped by both RADseq and GT‐seq and tissue samples genotyped by both WGS and GT‐seq.

We calculated genotyping discordance by extracting filtered (>50% missing data) GT‐seq loci from the RADseq data and comparing genotypes at those loci for 53 individuals that were sequenced with both RADseq and GT‐seq. In Barbosa et al. (2021), RADseq SNPs were genotyped using the multinomial maximum‐likelihood framework STACKS (Catchen et al., 2013), and VCFTOOLS (Danecek et al., 2011) was used to exclude individuals with ≥50% missing data and include biallelic SNPs with <50% missing data located >10,000 bp apart with >2% minor allele frequency and >3 reads. This genotyping pipeline will be referred to as the maximum‐likelihood (ML) pipeline. First, we calculated genotyping discordance between GT‐seq data derived from the AR pipeline and RADseq data derived from the ML pipeline. To remove discrepancies in discordance calculations due to differences in genotyping calling methods, we also used the AR pipeline to genotype the RADseq data and then calculated discordance between GT‐seq and RADseq data derived from the AR pipeline (https://github.com/mjgarrett/NIDGS_scripts).

Twenty‐one NIDGS tissue samples were genotyped at an average of 7× coverage with WGS and also by the GT‐seq panel. The same set of loci used in the RADseq comparison was extracted from the WGS data and used to calculate discordance for the genotypes produced from the tissue samples between WGS and GT‐seq.

### 2.6 Population genetic analyses

We compared the results from genetic diversity and population structure analyses produced by GT‐seq and RADseq for the 53 individuals sequenced with both methods. We selected only neutral loci to measure and compare estimates of these demographic processes across methods. We created three datasets: all 2663 (filtered) neutral SNPs identified and used in the RADseq study (hereafter referred to as RAD_2663), the 207 neutral SNPs used and genotyped in the GT‐seq panel and pipeline (hereafter referred to as GT_207), and the same 207 GT‐seq loci extracted from the RADseq sequencing data (hereafter referred to as RAD_207).

We performed analyses for sites with ≥8 individuals for all three datasets. From R package HIERFSTAT (Goudet, 2005), *H*_O_, *H*_E_, and *F*_IS_ were estimated with function *basic.stats*, and pairwise *F*_ST_ was estimated with functions *genet.dist* and *boot.ppfst* with 999 bootstraps.

Population structure was evaluated using two approaches: Bayesian clustering analysis as implemented in program STRUCTURE (Pritchard et al., 2000) and maximum likelihood clustering analysis as implemented in program ADMIXTURE (Alexander et al., 2009). For STRUCTURE, we applied an admixture model with a burn‐in period of 100,000 and a run length of 500,000 MCMC replicates to determine the number of genetic clusters (*K*) from 1 to 10 over 10 iterations each. The optimal *K* for each dataset was identified according to the Evanno method (Evanno et al., 2005) and the rate of change in the likelihood values (Pritchard et al., 2000). For ADMIXTURE, we used the *admixture‐wrapper.py* python script (https://github.com/dportik/admixture‐wrapper) to find the optimal *K* from 1 to 10 which was run over 50 repetitions and determined from its cross‐validation procedure, which fold number was set to 10. Both STRUCTURE and ADMIXTURE outputs were visualized with the following R script (https://github.com/sakura81/PYRA_Genomics).

## 3 RESULTS

### 3.1 GT‐seq panel development

From the 2663 neutral and 131 putatively adaptive NIDGS SNPs discovered in the Barbosa et al. (2021) RADseq study, primers were designed for 510 neutral and all 131 putatively adaptive SNPs (Table 1). After the first optimization, 55 neutral and 13 adaptive SNPs were removed due to primer artifacts, off‐target amplicons, and overamplification of the target sequence which can indicate multiple genomic copies of the target amplicon. The second optimization began with a panel of 573 SNPs, consisting of 455 neutral and 118 adaptive SNPs. After the second optimization, 231 neutral and 37 adaptive SNPs produced primer artifacts, off‐target amplification, and over amplification of the target amplicon. In the third optimization, the standard mix with equal concentrations for each primer had a higher genotyping success than the variable primer mix, 82.6% and 74.2% respectively. Therefore, the optimized GT‐seq panel includes primers for 305 SNP loci (224 neutral and 81 adaptive SNPs).

**TABLE 1:** NIDGS GT‐seq panel optimization with the selection of SNPs from the Barbosa et al. (2021) RADseq study, primer development, optimization, and the composition of the final NIDGS GT‐seq panel.

	Neutral	Adaptive	Total
RADseq NIDGS SNPs	2663	131	2794
Primer development	510	131	641
First optimization			
Candidate SNPs	510	131	641
SNPs removed	55	13	68
Second optimization			
Candidate SNPs	455	118	573
SNPs removed	231	37	268
Final GT‐seq NIDGS panel	224	81	305

### 3.2 Sample quality and GT‐seq genotyping success

Qubit concentration for hair samples and buccal swabs ranged from >0.05–1.62 to 0.78–36.7 ng/μL, respectively. For the hair extracts, gel electrophoresis revealed poor DNA quality, and gDNA fragment analysis reported DNA concentrations from 0.0132 to 0.873 ng/μL. Forty‐seven out of 53 hair extracts amplified one microsatellite locus.

Sequencing yielded 112,211 mean reads per individual (range = 1556–4,377,287) for hair samples, 201,904 reads per individual (range = 10–18,205,105) for buccal swabs, and 3,392,926 reads per individual (range = 120,622–12,277,944) for tissue samples. Hair samples yielded 1552 mean on‐target reads per individual (range = 3–107,011), buccal swabs yielded 116,184 mean on‐target reads per individual (range = 1–11,219,616), and tissue samples had 1,330,799 mean on‐target reads per individual (range = 10,104–4,992,869).

**FIGURE 2:**
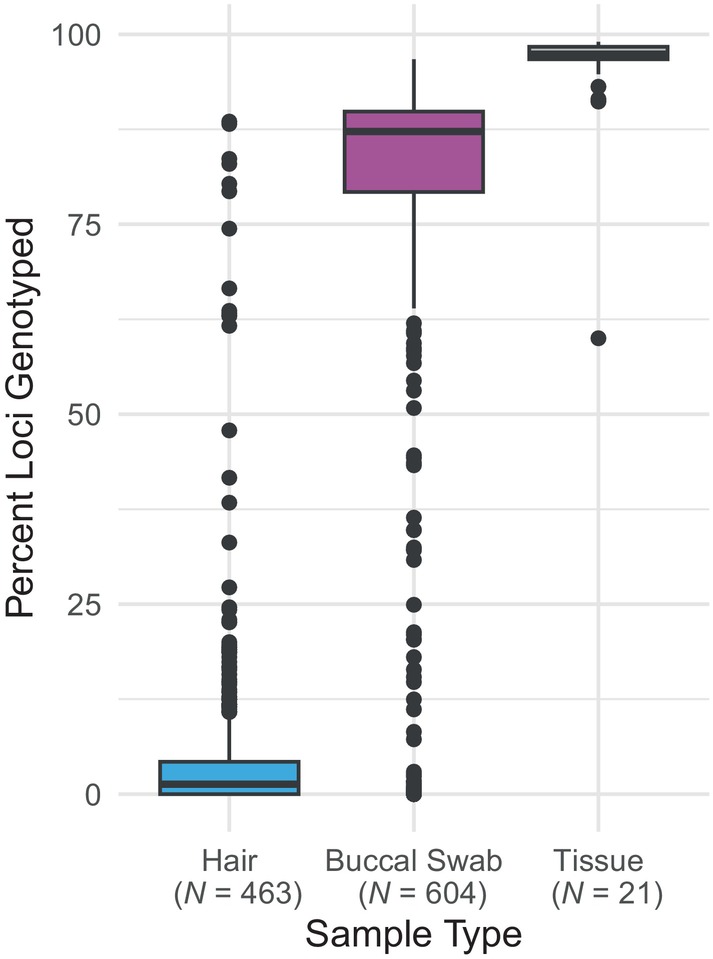
Boxplot of percent loci genotyped by sample type.

After we filtered out loci that had no data from 50% or more of our samples, 280 SNPs remained in the GT‐seq dataset. The percentage of loci genotyped from hair samples was lower than buccal swabs and tissue samples (Figure 2). The average percentage of loci genotyped differed among the three sample types: 5.55 for hair samples (min = 0, max = 88.52, median = 1.31), 73.16 for swab samples (min = 0, max = 96.72, median = 87.21), and 95.48 for tissue samples (min = 60, max = 99.02, median = 97.38).

### 3.3 Genotyping discordance

When we compared the GT‐seq data derived from the AR pipeline to the RADseq data derived from the ML pipeline, we used 280 SNPs and 53 shared individuals. We found a discordance rate of 7% (Table 2). Of the discordant SNPs, 77% have a homozygous genotype from RADseq and a heterozygous genotype from GT‐seq. For the GT‐seq data and the RADseq data both genotyped with the AR pipeline, 274 SNPs were used to compare the same 53 individuals, as six SNPs were removed in the re‐calling of the RADseq data with the AR pipeline. For the GT‐seq and RADseq data derived from the AR pipeline, the discordance rate is 4%. Of the discordant SNPs, 66% have a homozygous genotype from RADseq and a heterozygous genotype from GT‐seq. The same 280 SNPs from the first comparison were used to evaluate concordance between WGS and GT‐seq tissue data, which has a discordance rate of 2%. Of the discordant SNPs, 40% have a homozygous genotype from WGS and a heterozygous genotype from GT‐seq. More information about concordance and types of discordance is in Table S2.

**TABLE 2:** Discordance between RADseq, WGS, and GT‐seq for 53 NIDGS buccal swab samples and 21 tissue samples.

Sequencing methods	Sample type	RADseq pipeline	SNPs	Mean number of loci per individual					
				Both methods	Discordant loci	RAD/WGS hom, GT het	RAD/WGS het, GT hom	RAD/WGS hom, GT hom	Discordance rate
RADseq vs. GT‐seq	Swab	ML	280	198.04	12.46	9.85 (77%)	1.87 (17%)	0.75 (6%)	7%
RADseq vs. GT‐seq	Swab	AR	274	97.37	3.19	2.10 (66%)	0.9 (22%)	0.19 (7%)	4%
WGS vs. GT‐seq	Tissue	—	280	213.55	4.7	1.85 (40%)	2.75 (53%)	0.10 (2%)	2%

*Note*: Discordance is a mismatch in diploid genotypes between datasets for the same individual. RADseq pipeline: the maximum likelihood (ML) or allele ratio (AR) genotyping pipeline was used to genotype RADseq data; Both methods: when RADseq and GT‐seq or WGS and GT‐seq call a locus; RAD/WGS hom, GT het: of the discordant loci, RADseq or WGS called a homozygote while GT‐seq called a heterozygote; RAD/WGS het, GT hom: of the discordant loci, RADseq or WGS called a heterozygote while GT‐seq called a homozygote; RAD/WGS hom, GT hom: of the discordant loci, RADseq or WGS and GT‐seq called a homozygote. Discordance rate calculated by dividing mean discordant loci per individual over loci genotyped by both methods. Percentages in previous categories are of discordant SNPs, while Discordance rate is of all SNPs.

### 3.4 Population diversity estimates

**FIGURE 3:**
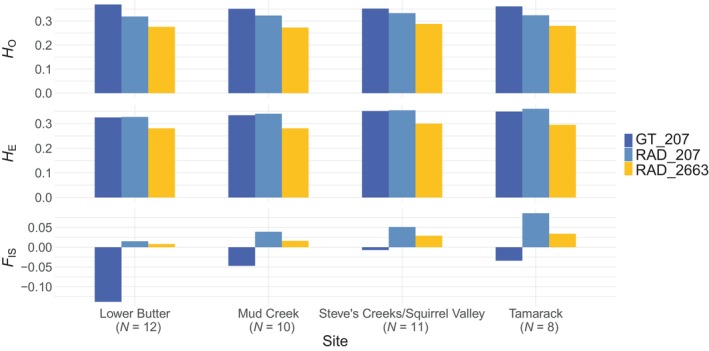
Population genetic diversity estimates of NIDGS by sequencing method for sites with more than eight individuals. RAD_2336 metrics estimated with 2336 neutral SNPs, RAD_207 and GT_207 with 207 neutral SNPs. *F*_IS_, inbreeding coefficient; *H*_E_, expected heterozygosity; *H*_O_, observed heterozygosity.

Overall, population diversity metrics are comparable across sequencing methods (Figure 3). RAD_2663 has the lowest *H*_O_ and *H*_E_ estimates, and RAD_207 has the highest *H*_E_ estimates. For both RADseq datasets, *H*_O_ < *H*_E_, while GT_207 suggested more heterozygotes than what would be expected under Hardy–Weinberg conditions. Thus, *F*_IS_ values for GT_207 are negative, and *F*_IS_ values are lower in RAD_2663 than RAD_207. Pairwise *F*_ST_ values are lowest in RAD_2263 and highest in GT_207 (Table 3).

**TABLE 3:** Pairwise *F*_*ST*_ (Weir & Cockerham, 1984) for NIDGS of sites with more than eight individuals across three datasets (RAD_2663, RAD_207, GT_207).

Dataset	LB vs. MC	LB vs. SS	LB vs. TA	MC vs. SS	MC vs. TA	SS vs. TA
RAD_2663	0.089 (0.081, 0.097)	0.080 (0.073, 0.088)	0.073 (0.066, 0.081)	0.094 (0.086, 0.101)	0.067 (0.060, 0.074)	0.0811 (0.074, 0.089)
RAD_207	0.093 (0.070, 0.120)	0.086 (0.062, 0.116)	0.074 (0.049, 0.099)	0.114 (0.089, 0.140)	0.071 (0.049, 0.140)	0.097 (0.069, 0.123)
GT_207	0.110 (0.080, 0.143)	0.094 (0.068, 0.123)	0.095 (0.070, 0.123)	0.129 (0.103, 0.157)	0.089 (0.064, 0.118)	0.110 (0.082, 0.142)

*Note*: Estimates were calculated with 999 bootstraps with confidence intervals for 0.025 and 0.975 given in parentheses.

**FIGURE 4:**
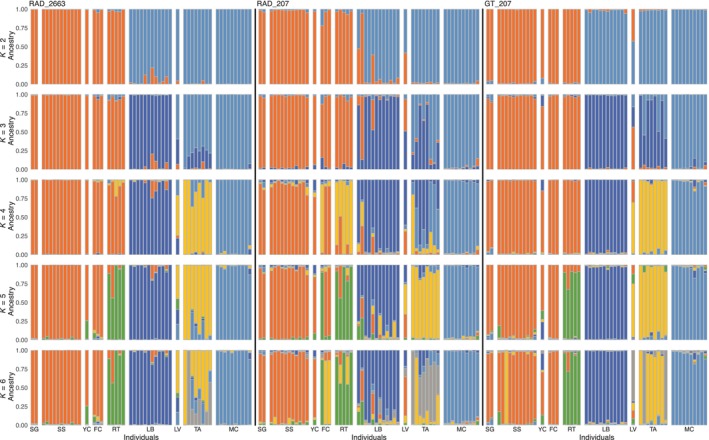
STRUCTURE bar plots displaying inferred clustering and individual ancestry estimates of NIDGS (*n* = 53) for all three neutral SNP datasets (RAD_2663, RAD_207, and GT_207) for *K*s 2–6. Each color represents a distinct genetic cluster, each vertical bar represents the proportion of ancestry of a single individual to the different genetic cluster. Individuals are grouped into populations which are ordered geographically from west to east: FC, Fawn Creek; LB, Lower Butter; LV, Lost Valley; MC, Mud Creek; RT, Rockytop; SG, Summit Gulch; SS, Steve's Creek/Squirrel Valley; TA, Tamarack; YC, YCC.

Patterns of proportion of assignment to a given *K* between programs STRUCTURE (Figure 4) and ADMIXTURE (Figures S5–S7) were consistent across *K* values and sequencing methods. However, among the STRUCTURE results, the best supported *K*s are different between sequencing methods, although there was not strong statistical support for a single “best” *K* for any of the datasets. For the RAD_2663 dataset, *K* = 6 is best supported (Figure S1), while *K* = 2 (with subpeak at *K* = 5) and *K* = 4 are best supported in RAD_207 (Figure S2) and GT_207 (Figure S3), respectively. Nevertheless, the genetic grouping patterns at each *K* among all three datasets are broadly consistent.

## 4 DISCUSSION

Here, we developed a GT‐seq panel of 305 loci for genetic monitoring of NIDGS than can be used on DNA extracted from minimally invasive buccal swab samples. We validated the GT‐seq panel by comparing genotypes and downstream genetic diversity and structure analyses across sequencing methods, and we evaluated factors that produced discordance between methods.

### 4.1 GT‐seq panel optimization

In spite of efforts to limit the potential for off‐target sequence capture and primer artifacts when designing primers for GT‐seq, 106 of the attempted 411 loci were excluded during testing of the panel. Typical conversion rates from designed primers to a functioning assay in GT‐seq panels average about 73% (Burgess et al., 2022; Chang et al., 2022; Hayward et al., 2022; McKinney et al., 2022; Schmidt et al., 2020) and for our study the rate was 74%. Of the filtered primer sets, 24 had no sequences reported for the target amplicon, 37 produced an abundance of off‐target sequences, three produced an overabundance of the target sequence, and the remaining 58 were omitted to prevent primer artifacts from forming in PCR or due to poor results in the plots produced by the *GTseq_SeqTest.pl* and *GTseq_Primer‐Interaction‐Test.pl* scripts. In this case, more loci than average were omitted for producing an abundance of off‐target sequences with properly paired primers. This means that some primer sets were targeting multiple genomic locations and were not efficiently capturing data from the target loci. This may be due to target locus sequences being repeated or present in transposable elements in the NIDGS genome. In Barbosa et al. (2021), RADseq data were aligned to the thirteen‐lined ground squirrel (*Ictidomys tridecemlineatus*) genome, as no NIDGS genome is currently available. This could be a possible reason many primer sets amplify in multiple regions, and this represents a general challenge for working with rare or threatened species where a reference genome is only available for a closely related species.

### 4.2 Performance of hair samples

The NIDGS hair samples performed poorly on GT‐seq. Despite producing similar raw read quantities to buccal swabs, hair samples yielded almost no on‐target reads. The hair samples were originally used in two studies that amplified eight nuclear DNA microsatellites, which ranged in 106–246 bp in size, to evaluate diversity and divergence in the NIDGS (Garner et al., 2005; Hoisington‐Lopez et al., 2012). When evaluated for their use in RADseq, it was determined the hair samples did not have high enough DNA quantities required by the technique. Despite the hair samples working in previous studies with microsatellite amplification, little to no DNA remained in the hair samples that we used for this study. These extracted DNA samples had been stored in a −80°C freezer after DNA extraction but had been through multiple freeze–thaw cycles which could have degraded the DNA. However, GT‐seq and other SNP panels have been successfully developed to be used with DNA extracted from hair samples in other species including Sitka black‐tailed deer, coyotes (*Canis latrans*), and tigers (*Panthera tigris*) (Burgess et al., 2022; Eriksson et al., 2020; Natesh et al., 2019).

### 4.3 Factors contributing to discordance

Two main factors likely explain genotype discordance: sequencing technique and genotyping method. This is the first study to design a GT‐seq panel from SNPs identified from RADseq performed on minimally invasive samples, rather than high‐quality samples like tissue or blood. RADseq with low coverage (e.g., <5× read depth) has a high probability of only sampling one chromosome of a diploid individual at a locus, which may cause heterozygotes to be called homozygotes in downstream analyses (Andrews et al., 2016; Nielsen et al., 2011). In comparing the ML pipeline derived and AR pipeline derived RADseq data to the AR pipeline derived GT‐seq data, 79% and 65% of discordant SNPs are from RADseq calling a homozygote and GT‐seq calling a heterozygote. If these homozygote‐heterozygote mismatch SNPs are removed, our discordance rate falls to 1.4% and less than 1%, in line with or below other studies (Hayward et al., 2022; Schmidt et al., 2020; Setzke et al., 2021). Moreover, in all genotyping pipeline comparisons, there are no heterozygote–heterozygote discordant SNPs, which supports the view that discordance is more often due to allelic drop‐out, particularly in lower‐coverage RADseq, than to detection of a false allele. In our data, GT‐seq provided higher coverage than RADseq overall, with GT‐seq having a mean coverage of 540.78× per locus, per individual and RADseq having a mean coverage of 9.65× per locus, per individual for the same 280 loci and 53 individuals. This is typical of how these two approaches are generally used because of the large difference in numbers of loci targeted and the trade‐off with total sequencing effort. A similar bias toward RADseq homozygous calls has also been found in other RADseq and SNP panel comparisons (Hess et al., 2015; Schmidt et al., 2020).

We found that genotype discordance is higher when calls are made from different genotyping pipelines. There is variability in the genotyping pipelines used in previous studies that compared RADseq and GT‐seq data. Schmidt et al. (2020) and Setzke et al. (2021) both used RADseq data called with the ML pipeline and GT‐seq data called with the AR pipeline. Bootsma et al. (2020) used GTscore (https://github.com/gjmckinney/GTscore) for GT‐seq genotyping and only retained GT‐seq genotypes if they had 60× coverage in their RADseq data, which was genotyped with the ML pipeline. Finally, Hayward et al. (2022) called their GT‐seq data multiple ways, first with the AR pipeline and then with the same ML approach as their double digest RADseq (ddRADseq) data. However, these studies have a RADseq and GT‐seq genotyping discordance rate between 1.1% and 3.4%. Our discordance rates for the tissue samples and the AR pipeline derived RADseq and GT‐seq data fall within this range. Thus, discordance is partly due to genotyping method, which is expected given how the choice and parameterization of genotyping methods is known to affect population genomic data (Mastretta‐Yanes et al., 2015; Paris et al., 2017; Rivera‐Colón & Catchen, 2022).

### 4.4 Genetic diversity and population structure

Our GT‐seq dataset produced similar patterns of genetic diversity and population structure to the RADseq dataset with one exception: population‐level *F*_IS_ values were consistently negative for the GT‐seq dataset. This finding agrees with other GT‐seq validation studies. Bootsma et al. (2020) and Schmidt et al. (2020) also report higher *H*_O_ than *H*_E_ values for GT‐seq data, as well as negative *F*_IS_ values. Positive *F*_IS_ values are often expected, resulting from both biological and methodological factors. Hidden population structure or excess relatedness in the set of sampled individuals can elevate observed heterozygosity relative to expectations based on treating the samples as representing a single population. Allelic drop‐out as a result of low or uneven coverage can also lead to positive *F*_IS_ values, when true heterozygotes are genotyped as homozygotes but there is no bias in which allele is observed. In contrast, negative *F*_IS_ values are harder to explain. One possible explanation is amplification of duplicate sequences or paralogs, causing sequence divergence at different parts of the genome to resemble heterozygosity. Our GT‐seq panel design and optimization aimed to remove any primer pairs that bind in multiple genomic locations, but it is possible that some remain. A second possibility is mis‐genotyping true homozygotes as heterozygotes at single loci. As described above, we did not observe any heterozygote–heterozygote discordance, suggesting that detection of a false allele that is not present in the population is unlikely. However, detection of a false allele that is present in other individuals in the population could result from factors like index hopping during library preparation, and this could elevate the frequency of observed heterozygotes. To test the possible effect of genotyping method, we used another genotyping pipeline GTscore (https://github.com/gjmckinney/GTscore) to genotype the 53 individuals sequenced with GT‐seq. GTscore called all the same genotypes as the original AR pipeline and thus resulted in the same higher *H*_O_ than *H*_E_ values for GT‐seq data and negative *F*_IS_ values. For details and results on GTscore, see Supporting Information. The negative *F*_IS_ values observed both here and in other GT‐seq studies warrant further investigation.

Despite any concerns about heterozygosity and individual genotypes, estimates of genetic population structure in NIDGS are broadly consistent when estimated with different sequencing methods and quantities of loci. The population structuring across *K* values between ADMIXTURE and STRUCTURE was also consistent (Figure 4 and Figures S5–S7). Nonetheless, population assignment appears cleaner (i.e., fewer individuals with admixed ancestry) in the GT_207 dataset compared to RAD_207. This is likely because although these datasets include the same 207 SNPs, the RAD_207 dataset has higher levels of missing data than GT_207 because of overall sequencing coverage (Table S2), resulting in greater uncertainty in genetic cluster assignment of some individuals in RAD_207. The clustering from the GT_207 dataset appears more similar to the RAD_2663 results (Figure 4), illustrating that the larger number of loci in a typical RADseq study can compensate for higher levels of missing data. The loci in the GT‐seq panel were chosen in part to allow delineation of neutral population structure, and our results show that they do successfully capture this signal compared to the full RADseq data. As a result, the GT‐seq panel promises to be effective for population clustering and ancestry assignment in future work.

### 4.5 Future applications

Our GT‐seq panel will be applied as a monitoring tool in future and ongoing sampling to address recovery objectives set by USFWS (2003), such as defining metapopulations and conservation units based on neutral and adaptive loci (Funk et al., 2012; Turbek et al., 2023) and estimating *N*_e_. The inclusion of both neutral and putatively adaptive loci is important for genetic monitoring practices and informing conservation action. Because of the high quality of DNA required by RADseq, Barbosa et al. (2021) provided a snapshot in time rather than a study of temporal change in genetic parameters for NIDGS. Our GT‐seq panel will be used to gauge the genetic health of NIDGS by monitoring changes in allele frequencies over time and in response to any future management actions, disturbances, or stochastic events (Flanagan et al., 2018; Schwartz et al., 2007; Shafer et al., 2015). As levels of gene flow were found to be variable among NIDGS sites (Barbosa et al., 2021), it will be important to assess how demographic processes change through time. Moreover, population persistence may be dependent on locally adapted genotypes for many of the isolated populations (Rundell & Price, 2009). Additionally, due to different evolutionary histories at each site, the differences in standing genetic variation and adaptive potential are important considerations for breeding or reintroduction programs (Flanagan et al., 2018; Hansen et al., 2012). This panel can help illuminate these factors as well as carry the genetic monitoring of NIDGS into the future as climate change and anthropogenic pressures may increase risks for this threatened species.

## AUTHOR CONTRIBUTIONS

**Molly J. Garrett:** Conceptualization (equal); data curation (lead); formal analysis (lead); investigation (lead); methodology (equal); project administration (equal); validation (lead); visualization (lead); writing – original draft (lead); writing – review and editing (equal). **Stacey A. Nerkowski:** Investigation (supporting); methodology (supporting); project administration (supporting); supervision (supporting); writing – review and editing (equal). **Shannon Kieran:** Formal analysis (supporting); investigation (supporting); writing – review and editing (equal). **Nathan R. Campbell:** Methodology (equal); software (lead); writing – review and editing (equal). **Soraia Barbosa:** Formal analysis (supporting); investigation (supporting); writing – review and editing (equal). **Courtney J. Conway:** Conceptualization (equal); funding acquisition (equal); project administration (supporting); supervision (equal); writing – review and editing (equal). **Paul A. Hohenlohe:** Conceptualization (equal); funding acquisition (equal); investigation (supporting); methodology (supporting); project administration (supporting); supervision (equal); writing – review and editing (equal). **Lisette P. Waits:** Conceptualization (equal); funding acquisition (equal); investigation (supporting); methodology (supporting); project administration (supporting); supervision (equal); writing – review and editing (equal).

### OPEN RESEARCH BADGES

This article has earned an Open Data badge for making publicly available the digitally‐shareable data necessary to reproduce the reported results. The data is available at https://github.com/mjgarrett/NIDGS_scripts.

## Supporting information


Data S1:

## Data Availability

SNP genotyping pipelines, concordance scripts, and the GT‐seq SNP dataset in .csv format are available on GITHUB (https://github.com/mjgarrett/NIDGS_scripts). VCF files from the RADseq data are available on DRYAD (https://doi.org/10.5061/dryad.sj3tx965c).
