# Supplementary material for: Development and validation of a GT‐seq panel for genetic monitoring in a threatened species using minimally invasive sampling
Source: Ecol Evol. 2024 May 20;14(5):e11321. doi: 10.1002/ece3.11321 (PMC11103765; doi:10.1002/ece3.11321)
Supplement: Supplementary file 1 — Data S1: [file ECE3-14-e11321-s001.docx]

**Development and validation of a GT-seq panel for genetic monitoring in a threatened species using minimally invasive samples**

Molly J. Garrett, Stacey A. Nerkowski, Shannon Rose Blair, Nathan R. Campbell, Soraia Barbosa, Courtney J. Conway, Paul A. Hohenlohe, Lisette P. Waits

**SUPPLEMENTARY INFORMATION**

**GTscore methods and results**

**Table S1**: Samples collected by site and GT-seq genotype success by sample type

**Table S2**: Full concordance table

**Table S3**: Population genetic diversity estimates by sequencing/genotyping method for 53 individuals by site and overall

**Figure S1**: Evanno method for RAD_2663

**Figure S2**: Evanno method for RAD_207

**Figure S3**: Evanno method for GT_207

**Figure S4**: ADMIXTURE CV plots for all datasets

**Figure S5**: ADMIXTURE barplots for RAD_2663, *K* 2-6

**Figure S6**: ADMIXTURE barplots for RAD_207, *K* 2-6

**Figure S7**: ADMIXTURE barplots for GT_207, *K* 2-6

**GTscore methods and results**

All scripts can be found here: <https://github.com/gjmckinney/GTscore>

AmpliconReadCoutner.pl was used read individual fastq files.

Read counts were adjusted passed on read correction factors in the primer probe file.

The polyGen function, a maximum likelihood algorithm, was used for genotyping.

GTscore results table: observed heterozygosity (H_O_), expected heterozygosity (H_E_), and inbreeding coefficient (F_IS_) for NIDGS sites between two GT-seq genotyping pipelines – GTseq, referred to as the AR pipeline (https://github.com/GTseq), and GTscore. N is sample size for each of the six sites sampled. Site abbreviations: SS, Steve’s Creek/Squirrel Valley; FC, Fawn Creek; MC, Mud Creek; TA, Tamarack; RT, Rocktop; LB, Lower Butter.

Table S 1. Samples collected by site and GT-seq genotype success for hair and buccal swab samples. Hair samples were collected in 2002 and 2006, and buccal swab samples were collected in 2016 and 2020. GT-seq genotyping success based on >50% loci genotyped per individual.

Table S 2. Concordance and discordance between RADseq, whole genome sequencing (WGS), and GT-seq for 53 swab samples and 20 tissue samples. RAD/WGS only: RADseq or WGS genotyped a locus while GT-seq did not; GT only: GT-seq genotyped a locus while RADseq/WGS did not; Both: both methods genotyped a locus; Negative concordance: both methods did not genotype a locus; Positive concordance: both methods got the same genotype; Discordant: a mismatch in diploid genotypes between the same individual; Discordance rate: discordance/both; Both hom: of the discordant loci, both methods called a homozygous locus; Both het: of the discordant loci, both methods called a heterozygous locus; RAD/WGS hom, GT het: of the discordant loci, RADseq or WGS called a homozygote and GT-seq called a heterozygote; RAD/WGS het, GT hom: of the discordant loci, RADseq/WGS called a heterozygote and GT-seq called a homozygote.

Table S 3. Population genetic diversity estimates for NIDGS by sequencing/genotyping method for 53 individuals by site (sites with more than 8 individuals). RAD_2336 metrics estimated with 2336 SNPs, RAD_207 and GT_207 with 207 SNPs. Abbreviations: H_O_, observed heterozygosity; H_E_, expected heterozygosity; F_IS_, inbreeding coefficient.

Figure S 1. Evanno method for NIDGS, dataset RAD_2663. (A) Mean L(K) over 10 runs for each K value. (B) Rate of change of the likelihood distribution calculated as L’K = L(K) – L(K – 1). (C) Absolute values of the second order rate of change of the likelihood distribution calculated according to: |L”K| = |L’(K+1) – L’(K). (D) ΔK calculated as ΔK = m|L”(K)|/s(L(K)].

Figure S 2. Evanno method for NIDGS dataset RAD_207. (A) Mean L(K) over 10 runs for each K value. (B) Rate of change of the likelihood distribution calculated as L’K = L(K) – L(K – 1). (C) Absolute values of the second order rate of change of the likelihood distribution calculated according to: |L”K| = |L’(K+1) – L’(K). (D) ΔK calculated as ΔK = m|L”(K)|/s(L(K)].

Figure S 3. Evanno method for NIDGS dataset GT_207. (A) Mean L(K) over 10 runs for each K value. (B) Rate of change of the likelihood distribution calculated as L’K = L(K) – L(K – 1). (C) Absolute values of the second order rate of change of the likelihood distribution calculated according to: |L”K| = |L’(K+1) – L’(K). (D) ΔK calculated as ΔK = m|L”(K)|/s(L(K)].


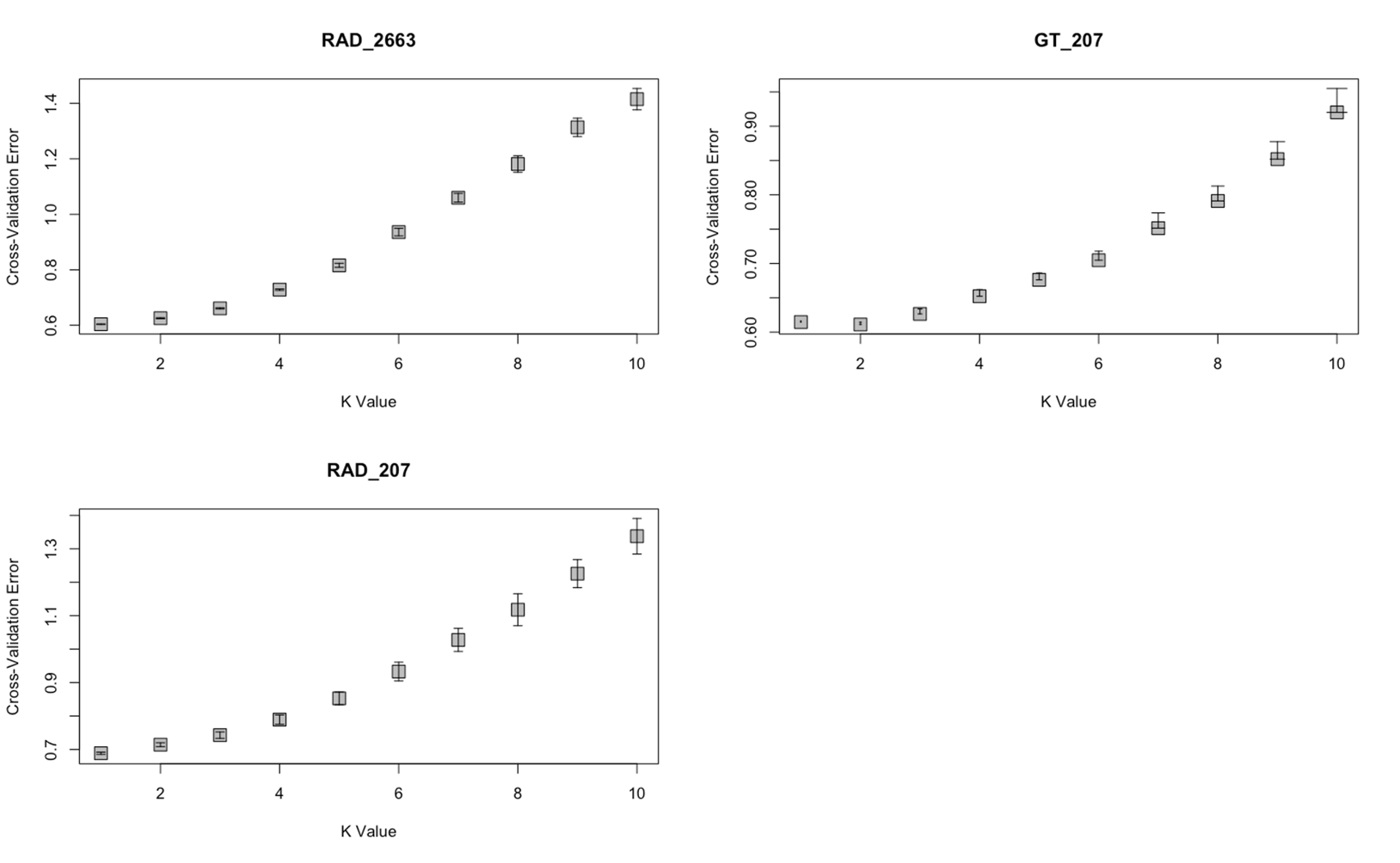


Figure S 4. Admixture cross-validation errors for K values from 1-10 for datasets RAD_2663, GT_207, and RAD_207.

Figure S 5. ADMIXTURE bar plots displaying inferred clustering and individual ancestry estimates of NIDGS (n = 53) for datasets RAD_2663 for K=2-6. Each color represents a distinct genetic cluster, each vertical bar represents the proportion of ancestry of a single individual to the different genetic cluster. Individuals are grouped into populations which are ordered geographically from west to east: SG, Summit Gulch; SS, Steve’s Creek/Squirrel Valley; YC, YCC; RT, Rockytop; LB, Lower Butter; LV, Lost Valley; TA, Tamarack; MC, Mud Creek.

Figure S 6. ADMIXTURE bar plots displaying inferred clustering and individual ancestry estimates of NIDGS (n = 53) for datasets RAD_207 for K=2-6. Each color represents a distinct genetic cluster, each vertical bar represents the proportion of ancestry of a single individual to the different genetic cluster. Individuals are grouped into populations which are ordered geographically from west to east: SG, Summit Gulch; SS, Steve’s Creek/Squirrel Valley; YC, YCC; RT, Rockytop; LB, Lower Butter; LV, Lost Valley; TA, Tamarack; MC, Mud Creek.

Figure S 7. ADMIXTURE bar plots displaying inferred clustering and individual ancestry estimates of NIDGS (n = 53) for datasets GT_207 for K=2-6. Each color represents a distinct genetic cluster, each vertical bar represents the proportion of ancestry of a single individual to the different genetic cluster. Individuals are grouped into populations which are ordered geographically from west to east: SG, Summit Gulch; SS, Steve’s Creek/Squirrel Valley; YC, YCC; RT, Rockytop; LB, Lower Butter; LV, Lost Valley; TA, Tamarack; MC, Mud Creek.
